# Rs2853677 modulates Snail1 binding to the *TERT* enhancer and affects lung adenocarcinoma susceptibility

**DOI:** 10.18632/oncotarget.9339

**Published:** 2016-05-13

**Authors:** Xiaoting Li, Xing Xu, Jiali Fang, Lin Wang, Yanchao Mu, Peng Zhang, Zhi Yao, Zhenyi Ma, Zhe Liu

**Affiliations:** ^1^ Department of Immunology, Biochemistry and Molecular Biology, 2011 Collaborative Innovation Center of Tianjin for Medical Epigenetics, Tianjin Key Laboratory of Medical Epigenetics, Tianjin Medical University, Tianjin, China; ^2^ Key Laboratory of Immune Microenvironment and Disease of the Ministry of Education, Tianjin Medical University, Tianjin, China; ^3^ Department of Forensic Medicine, Tianjin Medical University, Tianjin, China; ^4^ Department of Clinical Laboratory, Tianjin Medical University Cancer Institute and Hospital, Tianjin, China

**Keywords:** rs2853677, Snail1, TERT, enhancer

## Abstract

Genome wide association studies (GWAS) have shown that SNPs in non-coding regions are associated with inherited susceptibility to cancer. The effect of one single SNP, however, is weak. To identify potential co-factors of SNPs, we investigated the underlying mechanism by which SNPs affect lung cancer susceptibility. We found that rs2853677 is located within the Snail1 binding site in a *TERT* enhancer. This enhancer increases *TERT* transcription when juxtaposed to the *TERT* promoter. The binding of Snail1 to the enhancer disrupts enhancer-promoter colocalization and silences *TERT* transcription. The high risk variant of rs2853677 disrupts the Snail1 binding site and derepresses *TERT* expression in response to Snail1 upregulation, thus increasing lung adenocarcinoma susceptibility. Our data suggest that Snail1 may be a co-factor of rs2853677 for predicting lung adenocarcinoma susceptibility and prognosis.

## INTRODUCTION

Cancer is frequently characterized by genetic alterations accompanied by epigenetic changes. These changes result in the aberrant activation and/or silencing of tumor related genes and confer premalignant epithelial cells with the capacity for unrestrained proliferation, resistance to cell death, evasion of immune destruction, and progression to frank malignancy [1–3]. Genetic alterations include somatic mutations, gene amplification, gene deletions, and SNPs. Whereas somatic mutations contribute to sporadically arising cancers, SNPs, which are found throughout the genome and can be inherited, play important roles in both sporadic and familial forms of cancer. A better understanding of the SNPs underlying the development of cancer is strongly needed to elucidate the etiology of the disease and to identify high-risk individuals for targeted screening and/or prevention.

GWAS have identified more than 150 loci associated with increased susceptibility to cancer, according to the Catalog of Published Genome-wide Association Studies [4]. Some SNPs are located in the protein-coding region and affect cancer susceptibility by changing protein function. Most SNPs, however, are located outside of known protein-coding sequences [5]. When present in the promoter region, SNPs can create or delete transcription factor binding sites and have appreciable effects on both gene transcription and protein yield [6, 7]. When present in the 3′ UTR region, SNPs can affect the binding affinity of miRNA [8]. However, how SNPs that are located in introns or inter-gene regions alter cancer susceptibility is largely uncharacterized.

Lung cancer is the leading cause of cancer-related death in most countries. Lung cancer associated SNPs were analyzed by GWAS in patients of various ethnic backgrounds. A Chinese group identified two intronic SNPs (rs2736100 at 5p15.33 and rs4488809 at 3q28) and two intergenic SNPs (rs753955 at 13q12.12 and rs12296850 at 12q23.1) with MAFs > 0.25 that are associated with lung cancer in a Chinese population [9]. Another two SNPs (rs2853677 at 5p15.33 and rs2741354 at 8q21.1) have been reported to be associated with lung cancer in Japanese and European populations, respectively [10, 11]. However, how these SNPs affect lung cancer susceptibility is completely unknown. Here, we investigated these 6 previously reported risk loci and confirmed that rs2853677 in the second intron of the *telomerase reverse transcriptase* (*TERT*) gene is associated with a high risk of lung adenocarcinoma in the Han Chinese population. A DNA fragment encompassing rs2853677 functions as an enhancer, which increases *TERT* transcription when colocalizing with *TERT* promoter. Snail1 binds to the enhancer, reconfigures the chromatin structure within the *TERT* gene, and represses *TERT* transcription. The high risk allele of rs2853677 disrupts the Snail1 binding site, causing derepression of *TERT* transcription in response to Snail1 upregulation. Our data implicate that rs2853677 may be a potential biomarker for prognosis in Snail1 associated cancer.

## RESULTS

### rs2853677 is associated with an increased risk of lung adenocarcinoma

We successfully genotyped 6 SNPs using SBE assay. All SNPs conformed to HWE in the controls (Table 1). After adjustment for gender and age, none of the SNPs reached statistical significance (Table 2).

**Table 1:** HWE tests in controls

SNP ID	HWE *P* value in controls
rs753955	0.62
rs2853677	0.63
rs2736100	0.58
rs4488809	1
rs2741354	0.71
rs12296850	0.056

**Table 2:** Associations between 6 SNPs with lung cancer risk

SNP	Genotype	Control, *n* (%)	Case, *n* (%)	OR	95% CI	*P*
rs753955	T/T	159 (47.2)	158 (40.4)	Reference	Referent	
	C/T	142 (42.1)	174 (44.5)	1.18	0.74–1.88	0.493
	C/C	36 (10.7)	59 (15.1)	1.74	0.87–3.47	0.116
	Dominant			1.29	0.83–2.00	0.26
	Recessive			1.6	0.84–3.07	0.15
rs2853677	T/T	143 (42.4)	137 (35)	Reference	Reference	
	C/T	157 (46.6)	194 (49.6)	1.56	0.97–2.51	0.66
	C/C	37 (11)	60 (15.3)	1.86	0.92–3.78	0.085
	Dominant			1.62	1.03–2.54	0.035
	Recessive			1.45	0.76–2.80	0.26
rs2736100	T/T	117 (34.7)	109 (27.9)	Reference	Reference	
	G/T	159 (47.2)	201 (51.4)	1.27	0.77–2.09	0.359
	G/G	61 (18.1)	81 (20.7)	1.29	0.69–2.42	0.421
	Dominant			1.27	0.79–2.05	0.32
	Recessive			1.12	0.65–1.93	0.69
rs4488809	T/T	100 (29.7)	115 (29.4)	Reference	Reference	
	C/T	168 (49.9)	179 (45.8)	0.98	0.58–1.63	0.927
	C/C	69 (20.5)	97 (24.8)	0.97	0.52–1.82	0.934
	Dominant			0.98	0.60–1.59	0.93
	Recessive			0.99	0.58–1.68	0.97
rs2741354	G/G	159 (47.2)	175 (44.8)	Reference	Reference	
	G/A	143 (42.4)	187 (47.8)	1.46	0.92–2.31	0.104
	A/A	35 (10.4)	29 (7.4)	0.83	0.37–1.86	0.644
	Dominant			1.33	0.86–2.06	0.2
	Recessive			0.69	0.32–1.50	0.35
rs12296850	A/A	171 (50.7)	210 (53.7)	Reference	Reference	
	G/A	148 (43.9)	159 (40.7)	1.03	0.66–1.62	0.894
	G/G	18 (5.3)	22 (5.6)	0.91	0.34–2.42	0.843
	Dominant			1.02	0.66–1.57	0.94
	Recessive			0.89	0.34–2.33	0.82

NOTE: Statistically significant (*P* < 0.0083) associations are in bold.

Logistic regression was adjusted for gender and age.

Following subgroup analysis stratified by tumor histology, rs2853677 was associated with adenocarcinoma in a dominant model [OR_CC/CT VS. TT_ = 2.33 (1.33–4.06), *P* = 0.0024] (Table 3). It was not associated with either squamous cell carcinoma or small cell lung cancer (SCLC). The other SNPs were not associated with any subtype of lung cancer (Table 3).

**Table 3:** Associations between SNPs with differential subtypes of lung cancer

SNP	Genotype	Squamous			SCLC			Adenocarcinoma		
		OR	95% CI	*P*	OR	95% CI	*P*	OR	95% CI	*P*
rs753955	T/T	Reference	Reference		Reference	Reference		Reference	Reference	
	C/T	1.05	0.50–2.19	0.903	0.81	0.38–1.72	0.578	1.03	0.60–1.77	0.922
	C/C	2.38	0.85–6.68	0.098	2.7	0.99–7.33	0.052	1.16	0.48–2.78	0.741
	Dominant	1.27	0.64–2.55	0.49	1.1	0.55–2.19	0.78	1.05	0.63–1.76	0.85
	Recessive	2.33	0.90–6.01	0.082	2.99	1.17–7.62	0.026	1.14	0.50–2.63	0.75
rs2853677	T/T	Reference	Reference		Reference	Reference		Reference	Reference	
	C/T	1.03	0.50–2.09	0.945	1.07	0.52–2.19	0.859	2.27	1.26–4.06	0.006
	C/C	1.15	0.36–3.68	0.811	0.89	0.26–3.02	0.858	2.55	1.12–5.78	0.025
	Dominant	1.05	0.53–2.07	0.89	1.04	0.52–2.07	0.92	**2.33**	**1.33–4.06**	**0.0024**
	Recessive	1.14	0.38–3.41	0.82	0.86	0.27–2.75	0.8	1.58	1.75–3.29	0.23
rs2736100	T/T	Reference	Reference		Reference	Reference		Reference	Reference	
	G/T	0.85	0.40–1.82	0.672	0.75	0.34–1.64	0.475	1.65	0.89–3.05	0.111
	G/G	0.77	0.28–2.07	0.602	0.84	0.32–2.19	0.715	1.74	0.83–3.63	0.142
	Dominant	0.83	0.40–1.70	0.61	0.78	0.37–1.61	0.5	1.67	0.94–2.99	0.08
	Recessive	0.85	0.36–2.04	0.72	0.99	0.42–2.31	0.98	1.26	0.68–2.33	0.47
rs4488809	T/T	Reference	Reference		Reference	Reference		Reference	Reference	
	C/T	1.41	0.61–3.25	0.426	0.67	0.30–1.48	0.319	1	0.55–1.81	0.99
	C/C	1.18	0.45–3.13	0.733	0.81	0.31–2.07	0.656	0.89	0.43–1.84	0.747
	Dominant	1.33	0.60–2.94	0.48	0.71	0.34–1.48	0.37	0.96	0.55–1.69	0.89
	Recessive	0.94	0.43–2.05	0.87	1.03	0.45–2.34	0.94	0.89	0.48–1.66	0.71
rs2741354	G/G	Reference	Reference		Reference	Reference		Reference	Reference	
	G/A	1.71	0.84–3.50	0.142	1.45	0.70–2.98	0.317	1.72	1.00–2.95	0.049
	A/A	1.56	0.48–5.06	0.458	1.3	0.37–4.49	0.681	0.79	0.28–2.23	0.663
	Dominant	1.68	0.85–3.32	0.13	1.42	0.71–2.82	0.32	1.53	0.91–2.57	0.1
	Recessive	1.2	0.39–3.68	0.75	1.1	0.33–3.61	0.88	0.61	0.23–1.66	0.33
rs12296850	A/A	Reference	Reference		Reference	Reference		Reference	Reference	
	G/A	1.36	0.67–2.74	0.391	1.42	0.70–2.87	0.33	0.67	0.39–1.15	0.148
	G/G	1.29	0.34–5.00	0.708	0.69	0.11–4.35	0.693	0.76	0.24–2.41	0.644
	Dominant	1.35	0.69–2.65	0.38	1.34	0.67–2.67	0.41	0.68	0.41–1.15	0.15
	Recessive	1.12	0.30–4.17	0.86	0.58	0.09–3.52	0.53	0.89	0.29–2.78	0.85

NOTE: Statistically significant (*P* < 0.0028) associations are in bold.

### The region encompassing rs2853677 functions as an enhancer of *TERT*

**Figure 1:**
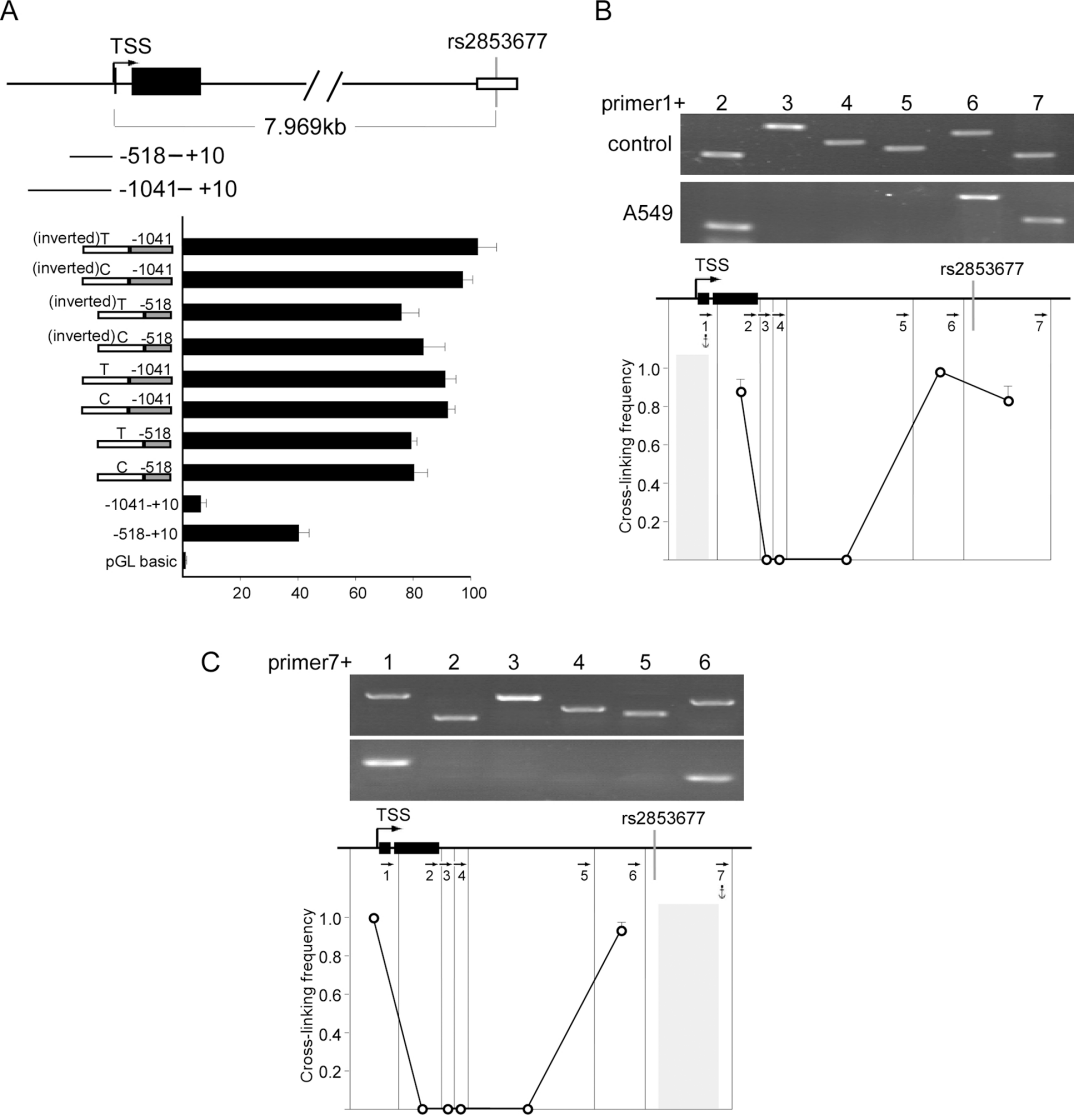
Rs2853677 is located inside of an enhancer (**A**) Luciferase reporter studies with serial extensions of the *TERT* promoter containing DNA fragment encompassing rs2853677 with T or C allele. The DNA fragment carrying T or C allele was amplified from A549 cells and placed directly upstream of the *TERT* promoter in forward or inverted orientation. The constructs were transiently transfected along with pRL-CMV *Renilla* luciferase reporter into HEK293 cells. luciferase activity was measured after 24 hrs' transfection. Mean ± SD indicates three independent transfections. (**B**) 3C was used to calculate cross linking frequency between chromatin segments to assess proximity in A549 cells. Vertical lines represent *Pst* I restriction sites. Arrows indicate PCR primer sites and direction. Anchor symbol marks anchoring primer. Cross linking frequency between the *TERT* promoter and different segments is shown. Top panels show representative PCR products. Mean ± SD of 3 independent chromatin preparations is shown. (**C**) 3C assay was performed with the DNA fragment harboring rs2853677 as an anchor fragment. Top panels show representative PCR products. Mean ± SD indicate 3 independent chromatin preparations.

Rs2853677 is located in the second intron at + 7969 base pairs (bp) from the transcription start site of the *TERT* gene. The *TERT* gene encodes the catalytic subunit of telomerase, the ribonucleoprotein complex that maintains telomere length. To test whether rs2853677 resides in a cis-regulatory element of *TERT*, a 600 bp fragment encompassing the rs2853677 was amplified from human A549 cellular DNA that carries the T/C genotype of rs2853677. PCR products with the T or C allele were inserted into a construct containing a luciferase reporter gene, upstream of the *TERT* promoter in the original or inverted orientation. Luciferase reporter constructs were transfected into HEK293 cells, and the − 518 to + 10 fragment exhibited basal promoter activity. Extension of the fragment to − 1041 repressed promoter activity, suggesting that a silencer exists within − 1041 to − 518 (Figure 1A). The DNA fragment encompassing rs2853677 increased expression of the reporter gene 2-fold when inserted directly upstream of the basic promoter (− 518–+ 10) and 10-fold when inserted directly upstream of − 1041–+10, in the forward or inverted orientation (Figure 1A). Both the T and the C allele exhibited the same effect on *TERT* promoter activity. These data suggest that rs2853677 resides in a transcriptional enhancer of *TERT*, whose function may be to abolish silencer activity.

We then explored whether the intronic enhancer encompassing rs2853677 is functional *in vivo*. Long range communication requires physical interaction between cis-regulatory elements with their target promoter [18, 19]. We used chromosome conformation capture (3C) to examine the physical proximity between the intronic enhancer and the *TERT* promoter. In brief, cross-linked chromatin was digested with *Pst*I, diluted and re-ligated, and long range association frequencies were assessed with PCR. We used Image Quant software to quantitate the PCR signals. The highest cross-linking frequency was set to 1.0 to normalize the variation between separate chromatin preparations. Very strong associations were detected between the *TERT* promoter and the intronic enhancer when the *TERT* promoter fragment was used as a PCR anchor in A549 cells (Figure 1B). Consistently, the same strong interaction between the intronic enhancer and the *TERT* promoter was detected when using the intronic enhancer as a PCR anchor (Figure 1C). Our data indicate that rs2853677 is located in a functional enhancer of *TERT*. Thus, the polymorphism of rs2853677 may affect the transcription of *TERT* by altering the binding affinity of a transcription factor.

### rs2853677-C disrupts a binding site for Snail1 and abolishes the suppressive role of Snail1 in *TERT* transcription

**Figure 2:**
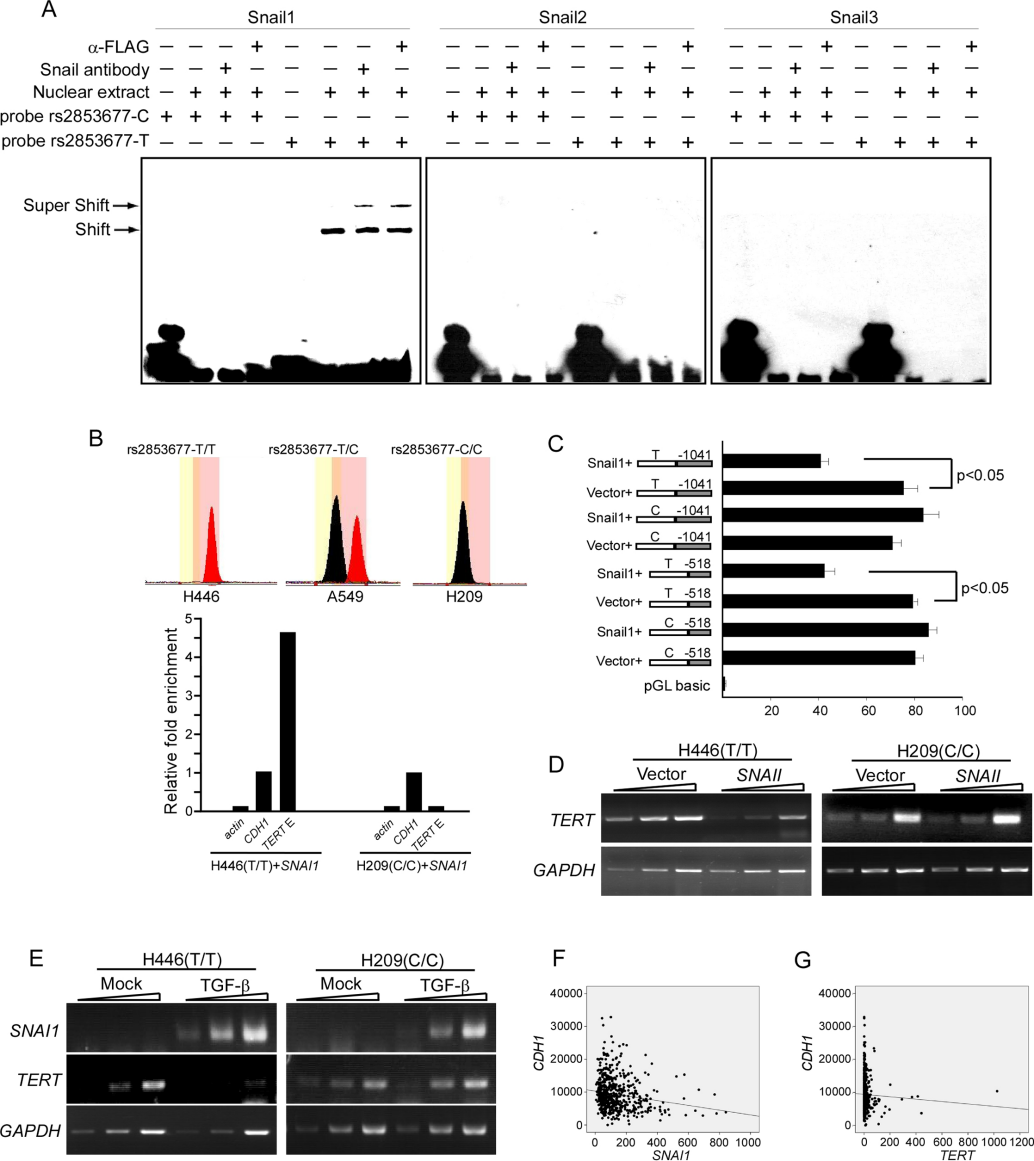
Rs2853677 affects *TERT* transcriptional regulation by Snail1 (**A**) EMSA shows mobility super shift (S.S.) of DNA fragment containing rs2853677. *SNAI1*-FLAG or *SNAI2*-FLAG or *SNAI3*-FLAG were overexpressed in H446 cells. Oligos carrying T or C allele were labeled with biotin in its 3′ terminus and incubated with nuclear extract that was purified from H446 expressing Snail1/2/3-FLAG. The antibody against Snail1/2/3 or FLAG was added to confirm the binding of Snail proteins to the oligos. Oligos harboring T allele but not the C allele can be bound by Snail1. (**B**) ChIP shows the occupancy of Snail1 in the *TERT* intronic enhancer *in vivo*. *SNAI1*-FLAG was overexpressed in H446 or H209 cells. ChIP was performed with FLAG antibody. Enrichment fold was evaluated by real-time PCR. Upper panels showed genotype of rs2853677 in H446 and H209 cells. Lower panels showed relative fold enrichment of Snail1 in the *TERT* intronic enhancer. *CDH1* promoter was used as a positive control and its enrichment was set to 1.0. Actin was used as a negative control. Association of Snail1-Flag with the *TERT* intronic enhancer was shown. (**C**) Luciferase reporter study shows effect of Snail1 cotransfection on the *TERT* intronic enhancer activity. Snail1 or empty vector was cotransfected with the enhancer-*TERT* promoter constructs into HEK293 cells. Luciferase activity was measured after 24 hrs' transfection. Mean ± SD indicate three independent transfections. (**D**) Semiquantitative RT-PCR was performed to measure *TERT* transcription in control or Snail1 expressing cells. 1 ul, 3 ul, 9 ul of reverse transcribed cDNA was analyzed by PCR. (**E**) Semiquantitative RT-PCR was performed to measure *TERT* and *SNAI1* transcription in control or TGF-β treated cells. 1 ul, 3u l, 9 ul of reverse transcribed cDNA was analyzed by PCR. (**F**) Expression of Snail1 and E-cadherin was assessed for correlation in lung adenocarcinoma using TCGA databases. *SNAI1* negatively correlated with *CDH1* (*r* = − 0.169, *p* = 4.545 × 10^−5^). (**G**) Expression of TERT and E-cadherin was assessed for correlation in lung adenocarcinoma using TCGA databases. *TERT* did not correlate with *CDH1* (*r* = − 0.041, *p* = 0.321).

Bioinformatic analysis revealed that CG[C/T]CTG is a potential binding site of the Snail family of proteins. We first evaluated the interaction between this consensus sequence and Snail proteins (Snail1, Snail2 and Snail3) and its modulation by rs2853677 *in vitro* using an EMSA assay. The 25 bp oligonucleotides encompassing the rs2853677-T or -C allele were labeled with biotin at their 3′ termini and incubated with nuclear extract extracted from Snail1-FLAG, Snail2-FLAG or Snail3-FLAG overexpressing H446 cells. Supershift assays were performed to confirm the association of the tagged Snail proteins with the respective oligonucleotide probes. A shifted band was observed when the oligos containing the T allele were incubated with nuclear extract containing Snail1-FLAG, but not Snail2-FLAG or Snail3-FLAG. The shifted band was supershifted when FLAG antibody or Snail1 antibody was added (Figure 2A). The C allele oligos, however, were not shifted by any nuclear extract (Figure 2A). This result indicates that Snail1 binds to the oligos carrying the T allele, but not the C allele. Neither Snail2 or Snail3 binds to either oligo. We then examined the effect of polymorphism rs2853677 on the *in vivo* occupancy of Snail1 to the consensus binding site using ChIP analysis. We analyzed the genotype of rs2853677 in 5 human lung cancer cell lines, including A549, H1229, H69, H209 and H446. H446 and H209, which carry the T/T and C/C genotypes, respectively, were selected for further analysis. Snail1 conjugated with FLAG at its C-terminus was transfected into these two cell lines. ChIP was performed using FLAG antibody. Enrichment of Snail1 in the *CDH1* promoter was set to 1.0. Occupancy of Snail1 with the *TERT* enhancer was detected in H446 cells, but not in H209 cells (Figure 2B). These data indicate that the rs2853677-C allele disrupts the Snail1 binding site.

We next tested whether this differential binding could modulate *TERT* gene expression in response to Snail1 upregulation. Luciferase assays of HEK293 cells showed that cotransfection of *SNAI1* with the luciferase reporter partially repressed enhancer activity when the enhancer harbored the T allele but had no effect on the enhancer carrying the C allele (Figure 2C), indicating that Snail1 inhibits enhancer activity upon association with the binding site. Accordingly, RT-PCR with one-, two-, or three-fold template DNA concentrations showed that transient expression of Snail1 repressed *TERT* transcription in H446 cells, but not in H209 cells (Figure 2D). Furthermore, *TERT* transcription was inhibited in H446 cells after 24 hr treatment with 10 μg/ml TGF-β, but not in TGF-β treated H209 cells, although both exhibited enhanced Snail1 expression upon TGF-β treatment (Figure 2E). These data indicate that Snail1 can bind to the enhancer and repress *TERT* transcription by inhibiting enhancer activity. The C allele of rs2853677 disrupts the association of Snail1 and prevents *TERT* downregulation in response to Snail1 upregulation.

The well-known function of Snail1 is to promote the epithelial-mesenchymal transition (EMT), a process that is critical for cancer malignancy and metastasis. We accessed The Cancer Genome Atlas (TCGA) public database to analyze any correlation among Snail1, TERT and E-cadherin (an important cell-cell adherent junction protein whose downregulation is a golden mark of the EMT) in lung adenocarcinoma. Expression of E-cadherin correlated negatively with Snail1 (*r* = − 0.169, *p* = 4.545 × 10^−5^), but not with TERT (*r* = − 0.041, *p* = 0.321) (Figure 2F). Accordingly, rs2853677 is not associated with lung adenocarcinoma metastasis [OR_CC/CT VS. TT_ = 0.090 (0.45–1.83), *P* = 0.78] (Table 4). These results suggest that the rs2853677-C allele has no effect on the EMT of lung adenocarcinoma.

**Table 4:** Association between rs2853677 and lung adenocarcinoma metastasis

Model	Genotype	OR (95% CI)	*P*
Codominant	T/T	Reference	0.6
	C/T	0.80 (0.38–1.69)	
	C/C	1.29 (0.47–3.50)	
Dominant	T/T	Reference	0.78
	C/T-C/C	0.90 (0.45–1.83)	
Recessive	T/T-C/T	Reference	0.4
	C/C	1.46 (0.60–3.60)	

NOTE: Logistic regression was adjusted for gender and age.

### Snail1 binds to the *TERT* enhancer and disrupts long range communication between the enhancer and the promoter

**Figure 3:**
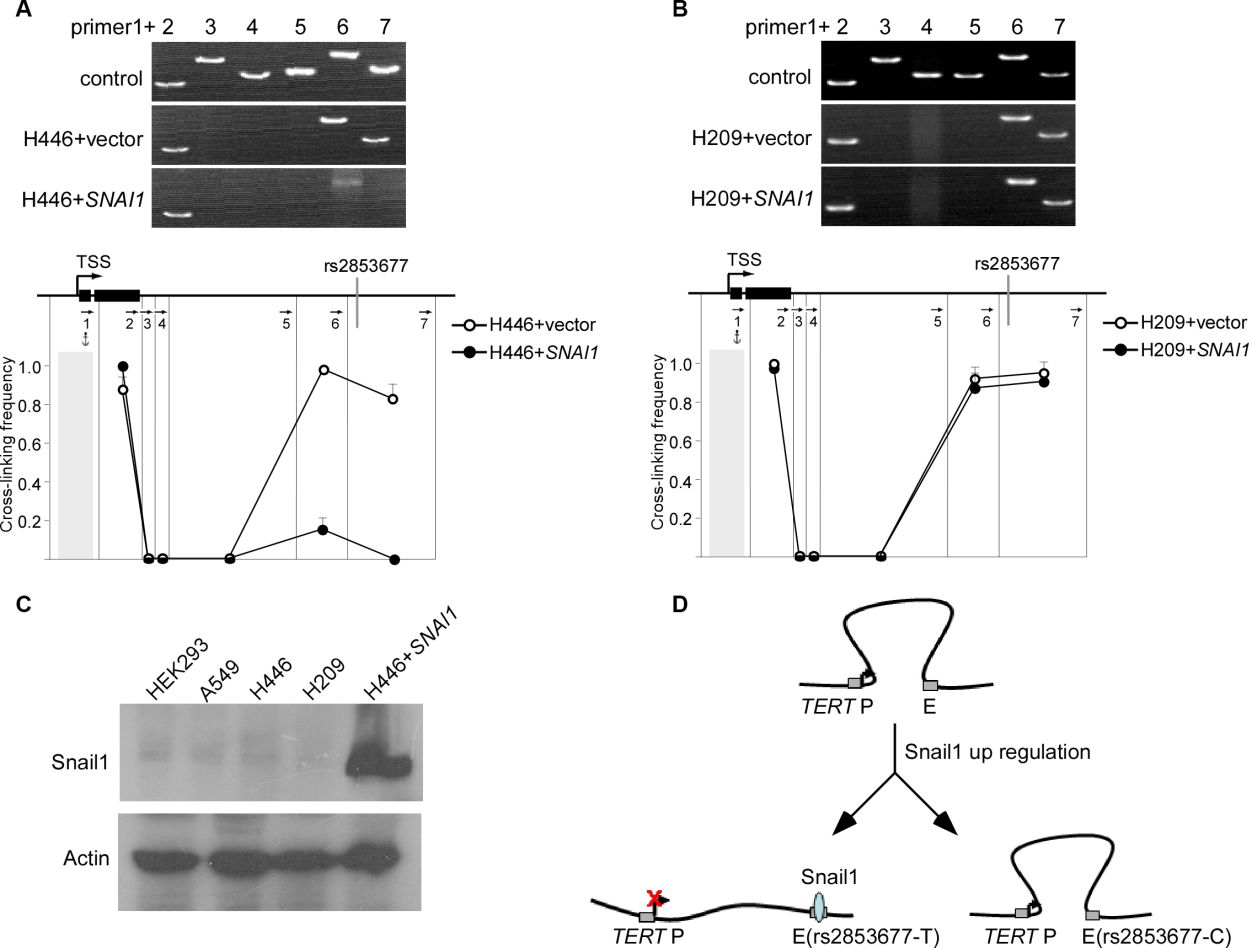
Snail1 disrupts long range communication between the intronic enhancer and the *TERT* promoter (**A**) Snail1 was overexpressed in H446 cells that harbor T/T genotype of rs2853677. 3C was performed to test the effect of Snail1 overexpression on proximity of the *TERT* enhancer and the promoter in H446 cells. Upper panels show representative PCR products. The lower panel shows the relative cross-linking frequencies between the *TERT* promoter and other DNA fragments. Mean ± SD indicate 3 independent chromatin preparations. (**B**) Snail1 was overexpressed in H209 cells that harbor C/C genotype of rs2853677. 3C was performed to test the effect of Snail1 overexpression on proximity of the *TERT* enhancer and the promoter in H209 cells. (**C**) Western blot showing the expression of Snail1 in variant cells. H446 expressing Snail1 was a positive control. (**D**) Schematic showing formation of a transcriptionally active complex containing the *TERT* promoter (*TERT* P) and the enhancer (**E**) Upon binding of Snail1 (shaded circles) to enhancer (T allele), the interaction is disrupted and *TERT* is downregulated.

We next investigated the mechanism by which Snail1 regulates the activity of the intronic enhancer. We have previously shown that the intronic enhancer physically interacts with the *TERT* promoter by looping out the intervening sequences. We examined whether Snail1 could change this chromatin structure using 3C. Transient expression of Snail1 caused delocalization of the intronic enhancer from the *TERT* promoter in H446 cells (Figure 3A), but not in H209 cells (Figure 3B). Notably, the *TERT* gene in both H446 and H209 cells exhibited physical interaction between the promoter and the intronic enhancer (Figure 3A and 3B), and the intronic enhancer carrying either the C or the T allele exhibited the same effect on *TERT* promoter activity in a luciferase assay in HEK293 cells. This leads us to postulate that Snail1 may not be expressed in these cells. We then examined the expression of Snail1 in HEK293, H446, and H209 cells. As expected, these three cell lines and A549 do not express Snail1 (Figure 3C). These data support a model whereby *TERT* transcription requires long-range physical interaction between the enhancer and the *TERT* promoter. Snail1 associates with the enhancer, disrupting enhancer-promoter physical interaction, downregulating *TERT* transcription. The C allele of rs2853677 disrupts the binding site of Snail1, thereby abolishing the suppressive effect of Snail1 on *TERT* transcription (Figure 3D).

## DISCUSSION

Genetic factors contribute to cancer susceptibility. Here, we confirmed that rs2853677, which is located in the second intron of *TERT*, is associated with a high risk of lung adenocarcinoma in the Han Chinese population. The high risk variant disrupts the Snail1 binding site and prevents repression of *TERT* in response to the upregulation of Snail1, thus resulting in a high level expression of *TERT* in the presence of Snail1.

TERT expression level determines telomere length, and overexpression of TERT immortalizes normal cells [20–22]. Several lines of evidence have shown that immortalized human cells are susceptible to transformation via the introduction of an oncogene [23–25]. There is also evidence showed that cotransfection of *TERT* with *H-ras* can transform immortalized cells [26]. Therefore, high levels of *TERT* expression in oncogene (for example, Snail1) expressing cells promote tumorigenesis. *TERT* promoter mutations at sites − 57, − 256, or − 228 are associated with melanoma, non-small cell lung cancer and bladder cancer, respectively [7, 27, 28]. Due to its importance, transcriptional regulation of *TERT* has been studied, and multiple cis-regulatory elements have been found within or upstream of the *TERT* promoter [29, 30]. In this report, we identified a new cis-regulatory element residing within the second intron. This cis-regulatory element attenuates silencer activity when juxtaposed to the *TERT* promoter by looping out the intervening DNA and increasing *TERT* transcription. An oncogenic transcription factor, Snail1, binds to the enhancer and disrupts this physical interaction, repressing *TERT* transcription.

Snail1 is a member of the Snail superfamily of zinc-finger transcription factors, which also includes Snail2 (Slug) and Snail3 (Smuc). Although the three members share a conserved DNA binding domain, and their regulated genes overlap, only Snail1 can bind to the sequence harboring rs2853677. Elevated Snail1 expression is detected in many types of cancer and correlates with tumor malignancy [31, 32]. Although Snail1 is generally considered to be an oncogene, our results clearly indicate that in addition to repressing the expression of E-cadherin, the enhanced expression of Snail1 also downregulates *TERT* transcription, which inhibits tumorigenesis. Therefore, Snail1 executes dual functions in tumorigenesis. The C allele of rs2853677, however, disrupts the Snail1 binding site and derepresses the expression of *TERT* by Snail1, blocking the inhibitory effect of Snail1 on tumorigenesis and thus increasing lung cancer susceptibility. Although the well-known function of Snail1 is to promote the EMT, rs2853677 does not seem to affect the EMT.

Unexpectedly, rs2853677 is associated only with lung adenocarcinoma and is not related to SCLC, although SCLC expresses similar levels of Snail1 compared with lung adenocarcinoma (Supplementary Figure S1) and SCLC expresses more TERT than lung adenocarcinoma [33, 34]. This may be due to other transcription factors or signaling pathways that are activated in SCLC and may block the effect of Snail1 on *TERT* transcriptional regulation. It is known that nearly all patients with SCLC are missing *RB1* and have more frequent mutations in *TP53* than patients with lung adenocarcinoma [35]. The p53 protein is a potent inhibitor of the *TERT* promoter [36]. Mutation of p53 can relieve repression of the *TERT* promoter and therefore lead to elevated TERT expression in SCLC. In addition, the PI3-kinase (PI3K) pathway, another positive *TERT* transcriptional regulator, is also activated in SCLC [37, 38]. The mechanism by which these signaling pathways activate the *TERT* promoter is not clear. It is possible that Snail1 induced *TERT* transcriptional inhibition is not feasible in the presence of these signaling pathways.

In summary, we identified Snail1 as a co-factor of rs2853677 for predicting susceptibility to and the prognosis of lung adenocarcinoma. Given that Snail1 expression is enhanced in many types of cancers, the association of rs2853677 with other cancer types needs to be studied.

## MATERIALS AND METHODS

### Cell lines

HEK293, A549, H1229, H69, H209, and H446 cell lines were from ATCC, and were cultured in RPMI1640 containing 10% FBS, 100 U of penicillin/ml, and 100 μg of streptomycin/ml.

### Study population

This case control study consisted of 391 lung cancer cases and 337 controls. The 391 patients with histologically confirmed lung cancer were recruited from Tianjin Medical University Cancer Institute & Hospital in China. The 337 controls were randomly selected from volunteers enrolled for physical examination at Tianjin General Hospital. All the controls did not have a history of cancer. Informed consent was obtained from all participants. This study was approved by Ethics Committee of Tianjin Medical University. The detail of the subject characteristics are listed in Table 5.

**Table 5:** Characteristics of cases and controls

	Controls, *n* (%)	Cases, *n* (%)	*P*
Total subjects	337 (100%)	391 (100%)	
Gender			0.969
Male	228 (67.7%)	264 (67.5%)	
Female	109 (32.3%)	127 (32.5%)	
Age			
Mean ± SD	38.8 ± 10.7	58.63 ± 8.8	0.000
Tumor type			
Squamous		107 (27.4%)	
SCLC		64 (16.4%)	
Adenocarcinoma		183 (46.8%)	
Other		37 (9.5%)	

### SNP selection and genotyping

6 previously reported risk loci located in the intron or inter-gene region were selected [9–14]. The information of six SNPs was listed in Table 6. The genotyping of 6 SNPs were examined in one PCR reaction. 2 μl of the PCR product was cleaned up with 1 μl of ExoSAP-IT (Amersham Biosciences, USA). Multiplex SBE reactions were performed in a total volume of 5 μL comprising 2 μL of SNaPshot ready reaction mix (Applied Biosystems, USA), 1.5 μL of cleaned PCR products and 1 μL of SBE primer mix. The SBE products were purified again using ExoSAP-IT. Genotyping for SNPs was performed by ABI 3130 Genetic Analyzer (Applied Biosystems, USA) according to the supplier's instructions. Primers were listed in Table 7.

**Table 6:** Information of six SNPs

SNPs	Allele	Locus	Location
rs753955	C/T	13q12.12	intergenic
rs2853677	C/T	5p15.33	intron of TERT
rs2736100	G/T	5p15.33	intron of TERT
rs4488809	C/T	3q28	intron of TP63
rs2741354	G/A	8q21.1	intergenic
rs12296850	G/A	12q23.1	intergenic

**Table 7:** Primers and probes used in experiment

primers used for genotyping of SNPs	
rs753955-F	5′-AATATAGGTGGGCCCTGTCC-3′
rs753955-R	5′-GGGAAAGACAATGCTGTGGT-3′
rs2853677-F	5′-CCAATCCAGTCTGACAGTCG-3′
rs2853677-R	5′-GAAACAAGGGAACGAGGACA-3′
rs2736100-F	5′-GTGCTGTTTTCCCTGCTGAC-3′
rs2736100-R	5′-GGGAACAAAGGAGGAAAAGC-3′
rs4488809-F	5′-ATGCAAGCATCTGCTCTTGA-3′
rs4488809-R	5′-TGTGCATTCCTGTGTTTCCT-3′
rs2741354-F	5′-GGCCAACACAAGGACTGACT-3′
rs2741354-R	5′-ATTTCGCTGCAGCTTCTTTC-3′
rs12296850-F	5′-AGGATTCATGGGATCAGTGG-3′
rs12296850-R	5′-GTAGGTCCCACAGGGAGTGA-3′
rs753955-SBE	5′- (GACT)1ATCATGTGAAGGCTTGAA-3′
rs2853677-SBE	5′- (GACT)3TTTGTCACTAGAGACCCG-3′
rs2736100-SBE	5′- (GACT)5TCCGTGTTGAGTGTTTCT-3′
rs4488809-SBE	5′- (GACT)7TGCTCTTGAGGCAGTAAA-3′
rs2741354-SBE	5′- (GACT)9GGTATCACCCTAAACCAAG-3′
rs12296850-SBE	5′- (GACT)10CACATATAAGTAAAAGGGCTTAC-3′
primers used for cloning	
SNAI1-CDS-F-Nflag	5′-CGGGATCCGCCACCATGGACTACAAGGACGACG ATGACAAGCCGCGCTCTTTCCTCGTCAGGA-3′
SNAI1-CDS-R	5′-CGGGATCCTCAGCGGGGACATCCTGAGCAGCCG GACTCTTGGT-3′
primers used for luciferase assay	
TERT-A	5′-AGGAAGCTTCGCTGCCTGAAACTCGC-3
TERT-S500	5′-TCACTCGAGGCATTCGTGGTGCCCG-3′
TERT-S1000	5′-GTCCTCGAGTGTCCTGCGGTTGTGCC-3′
SNP-F	5′-CGGGGTACCATGTGAGGCATTGTTAGGTGCAT-3′
SNP-R	5′-CGGGAGCTCCTAAGTCAGCAGGGAAAACAGCA-3′
primers used for EMSA	
Snail-EMSA-F1-biotin	5′-ACTAGAGACCCGCCTGGTGCACTCTG-biotin
Snail-EMSA-R1-biotin	5′-CAGAGTGCACCAGGCGGGTCTCTAGT-biotin
Snail-EMSA-F2-biotin	5′-ACTAGAGACCCGTCTGGTGCACTCTG-biotin
Snail-EMSA-R2-biotin	5′-CAGAGTGCACCAGACGGGTCTCTAGT-biotin
primers used for ChIP	
ChIP-S	5′-ACTCTTGGACTCAAGGGATC-3′
ChIP-A	5′-ACACTCGGCAGGAAACG-3′
E-Cad-S	5′-CAACTCCAGGCTAGAGGGTCACCGC-3′
E-Cad-A	5′-AACTGACTTCCGCAAGCTCACAGG-3′
β-actin-S	5′-GCCAACCGCGAGAAGATGACCCAGA-3′
β-actin-A	5′-GAGTCCATCACGATGCCAGTAG-3′
primers used for RT-PCR	
TERT-Q-F	5′-CCGATTGTGAACATGGACTACG-3′
TERT-Q-R	5′-CACGCTGAACAGTGCCTTC-3′
SNAI1-Q-F	5′-ACTGCAACAAGGAATACCTCAG-3′
SNAI1-Q-R	5′-GCACTGGTACTTCTTGACATCTG-3′
GAPDH-F	5′-GTCAACGGATTTGGTCGTATT-3′
GAPDH-R	5′-AGTCTTCTGGGTGGCAGTGAT-3′
primers used for 3C	
3C-1	5′-CCAGCCCCTCCCCTTCCTTT-3′
3C-2	5′-GCTCCAGGCACAACGAACGC-3′
3C-3	5′-GGAAGATGAGCGTGCGGGACT-3′
3C-4	5′-CGGTGGCTCACGCCGGTAAT-3′
3C-5	5′-CCCCAGGTGTCCTTGGCGTTTG-3′
3C-6	5′-GTTTGGTAGCATTTATGTGAGGCATTG-3′
3C-7	5′-TGTGCGGTGTCTGGATGG-3′
control 1-S	5′-GGGGAACCAGCGACATGC-3′
control 1-A	5′-CCCTGAACACCCACAAACACT-3′
control 2-S	5′-GCTTGCTCCTGAATGTTTGCT-3′
control 2-A	5′-TGGTGAGAAACAAGGGAACGAG-3′
control 3-A	5′-CTCCATACATCCAGCTCACC-3′
control 3-S	5′-TGGGAACCAGGACAAAGG-3′

### Data analysis

Differences of gender and age between cases and controls were compared by χ2 tests and student *t* tests respectively. Hardy-Weinberg Equilibrium (HWE) was assessed in the control samples by applying an exact test and then genotype frequencies were compared using χ2 tests. The association between lung cancer and each SNP was examined using logistic regression under Codominant, dominant and recessive models after adjusting for sex and age. The significance level was set at 0.018. These analyses were carried out using SNPStats [15] and SPSS 16.0.

### Cloning and transduction

Human *SNAI1* was amplified from H1155 cDNA. *SNAI2* or *SNAI3* was amplified from Beas-2B cDNA. FLAG was conjugated to the C-terminus. *SNAI1*-FLAG or *SNAI2*-FLAG or *SNAI3*-FLAG was ligated into the lentiviral shuttle pCCL.PPT.hPGK.IRES.GFP/pre. The plasmid was used to produce lentivirus in 293T cells with the packaging plasmids pMD2.BSBG, pMDLg/pRRE and pRSV-REV.

### Luciferase assay

A 528 bp and 1051 bp DNA fragment upstream of *TERT* TSS were amplified from A549 cells DNA using primers listed in Table 7. The DNA fragments were inserted into the pGLbasic. Two analogous 600 bp DNA fragments encompassing the rs2853677 site were amplified from A549 cells and the T or the C-allele were subsequently inserted in forward or inverted orientation into upstream of the *TERT* promoter. Plasmids were cotransfected into HEK293 cells using Lipofectamine 2000 (Invitrogen) with pRL-CMV Renilla luciferase reporter, which was used for normalization (Promega).

### Chromatin immunoprecipitation

*SNAI1*-FLAG was overexpressed in H446 and H209 cells. ChIP was performed using M2-argarsose as described previously [16]. Fold enrichment was analyzed by PCR assays. β-actin promoter was treated as a negative control and *CDH1* promoter was treated as a positive control. Primers used in this study are listed in Table 7.

### EMSA

Nuclear proteins from H446 expressing Snail1 or Snail2 or Snail3 cells were extracted using a NucBuster protein extraction kit (Novagen) according to the manufacturer's instructions. Double-stranded oligonucleotides corresponding to the potential Snail1 binding sites 5′-ACTAGAGACCCG[C/T]CTGGTGCACTCTG was end-labeled with biotin in its 3′ terminus. Binding assays were performed in 10 μl of reaction mixture with 2 μg of nuclear protein extracts and 1 nM labeled probes at room temperature for 30 min in binding buffer (10 mM Tris-Cl, 55 mM KCl, 2.5 mM MgCl_2_, 0.25 mM EDTA, 1 mM DTT, 0.05% NP-40, 5% Glycerol and 1 μg poly dI-dC). In supershift assay, Snail1 or Snail2 or Snail3 antibodys or FLAG antibody was added. Reactions were analyzed by electrophoresis on a 6.0% non-denaturing polyacrylamide gel at 100 V for 1 h. After transfer, the membrane was immediately cross-linked and bands detected by chemiluminescence (Pierce, USA).

### Chromosome conformation capture

3C assays were performed as described previously [17]. Briefly, 10^6^ cells were cross-linked, lysed, and nuclei were digested with *Pst* I. After ligation and subsequent DNA purification, the cross-linking frequencies between the anchor and test fragments were estimated by PCR reactions using primers listed in Table 7. To create a standard for normalization of relative PCR efficiencies, three PCR products together containing all the sequence from the *TERT* gene TSS to10 Kb downstream were amplified, digested with *Pst* I, and ligated at high concentrations to generate equimolar mixtures of all possible ligation products. The cross-linking and ligation efficiencies between different samples were normalized by setting the highest cross-linking frequency to 1.0.

## SUPPLEMENTARY MATERIALS FIGURE



## Abbreviations

The abbreviations used are

SNP: single nucleotide polymorphism
GWAS: genome-wide association studies
TERT: telomerase reverse transcriptase
HWE: Hardy-Weinberg Equilibrium
SBE: single base extension
3C: chromosome comformation capture
ChIP: chromatin immunoprecipitation
EMT: epithelial-mesenchymal transition
RT-PCR: reverse transcription-polymerase chain reaction
SCLC: small cell lung cancer
