# Supplementary material for: Rs2853677 modulates Snail1 binding to the TERT enhancer and affects lung adenocarcinoma susceptibility
Source: Oncotarget. 2016 May 13;7(25):37825–38. doi: 10.18632/oncotarget.9339 (PMC5122352; doi:10.18632/oncotarget.9339)
Supplement: Supplementary file 1 [file oncotarget-07-37825-s001.pdf]

Rs2853677 modulates Snail1 binding to the *TERT* enhancer and affects lung adenocarcinoma susceptibility

Supplementary Materials

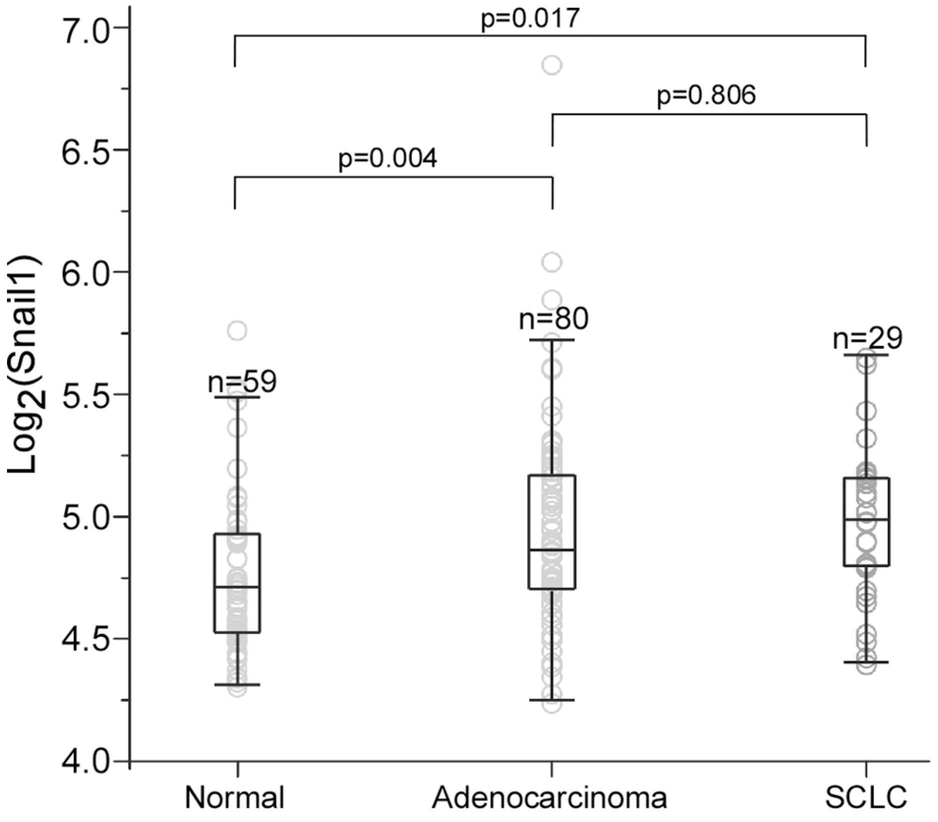

Supplementary Figure S1: Snail1 expression in lung adenocarcinoma cell lines and in SCLC cell lines. Gene Expression Ominubus database (GSE32036) was interrogated for Snail1 expression in human normal lung epithelial cells (59), lung adenocarcinoma cell lines (80) and SCLC cell lines (29) [39].
